# Advances in Pediatric Cardiology Nutrition

**DOI:** 10.3390/nu15122653

**Published:** 2023-06-07

**Authors:** Marcello Lanari, Laura Andreozzi, Marianna Fabi

**Affiliations:** Pediatric Emergency Unit, IRCCS Azienda Ospedaliero Universitaria di Bologna, 40138 Bologna, Italy; marcello.lanari@unibo.it (M.L.); laura.andreozzi4@unibo.it (L.A.)

The relationship between nutrition and cardiovascular diseases is powerful and complex. Nutritional interventions are crucial in patients diagnosed with cardiovascular diseases from a preventive, therapeutic and prognostic point of view. This interplay is more prevalent during childhood when significant physical growth occurs, thus affecting other aspects of children’s development, including their cognition, social behavior and personality.

Children diagnosed with both acute and chronic cardiovascular conditions need specific nutritional indications and care. Although great progress has been made in this area, several aspects still need to be clarified.

This Special Issue of Nutrients brings together several outstanding contributions, including original papers, brief reports and literature reviews, to explore different aspects of the complex relationship between nutrition and cardiovascular diseases in children and adolescents. This Editorial is intended to provide an overview of the articles collected in this Special Issue, leaving it to the reader to delve into this topic and explore each article individually.

Children with congenital heart disease (CHD) are the most representative population with nutritional issues in the pediatric age, both before and after possible surgical intervention, which affects both their short- and long-term outcomes. These children are considered part of a nutritional high-risk group, being at higher risk of undernutrition and failure to thrive. Malnutrition in this group population leads to a longer hospital stay and higher risk of infection, mortality rates, family stress and adverse neurodevelopmental outcomes [1,2].

The correlation between CHD and impaired nutritional status is well reported in low-income countries, where malnutrition is a social problem and corrective heart surgery often occurs later in life. In high-income countries, children usually receive an early diagnosis and undergo corrective surgery ahead of time. Additionally, proper nutritional interventions (including the use of increased calorie formulas and feeding tubes) are systematically offered to these patients. Early diagnosis and proper treatment appear to have reduced, but not removed, the impact of CHD on nutritional status. Palleri et al. conducted a comparative retrospective cross-sectional study, aiming to define the impact of CHD on the growth of infants and children in a high-income European country [3]. The authors showed that moderate and severe congenital heart diseases negatively affected the growth of children compared to the general population even in a tertiary center in a high-income country, where surgical interventions are performed early in life and proper nutritional support is guaranteed. On the other hand, patients with mild CHDs presented anthropometric parameters similar to those of healthy children. Finally, the authors found that the prevalence of obesity among children with CHD is comparable to that of the general population, exposing them to the risk of cardiovascular complications later in life.

A particular group of CHD patients at high risk of nutritional issues are those undergoing Fontan circulation (FC) palliation. FC is a surgical palliation performed on patients with various CHDs that may differ anatomically but are functionally characterized by an univentricular heart. Specific guidelines on the nutritional management of FC patients are still lacking despite the fact that malnutrition has significant implications for survival, quality of life and short- and long-term complications in these children. Baldini et al. conducted an extensive review on the nutritional management of these patients [4]. The authors reported the current evidence-based literature on the nutritional interventions in these patients, from the assessment of the nutritional status to the dietary interventions that should be taken into account throughout the different stages of these children’s lives. In addition, a practical and systematic approach is suggested to help physicians carry out an appropriate nutrition assessment, detect faltering growth early and adequately set up a nutritional intervention.

The papers by Palleri et al. and Baldini et al. definitively show that proper nutritional management is crucial in children with CHDs [3,4]. Samanidis et al. investigated another aspect of the complex relationship between nutrition and CHD, particularly the role of nutritional interventions in the management of postoperative complications [5]. The authors’ extensive narrative review summarizes the current evidence on the diagnosis and management of postoperative chylothorax in neonates and infants after CHD surgery, focusing on nutritional therapies. The rationale behind conservative nutritional management strategies, including either a combination of nil per os and total parenteral nutrition or enteral feeding with a medium-chain triglyceride diet, fat-modified breast milk or low-fat diet, is described.

Palleri et al., Baldini et al. and Samanidis et al. show the need for a shared consensus on the optimal nutritional management of children with CHDs at every stage of these patients’ lives, given the crucial role of nutritional status in this broad spectrum of diseases [3,4,5].

Like congenital cardiac malformations, atherosclerotic cardiovascular morbidity can begin early in life. While myocardial infarction, stroke and peripheral vascular disease usually affect adults, atherosclerosis is a silent killer that can start early in childhood, leading to the clinical manifestations of atherosclerotic cardiovascular disease later on. Early obesity and dyslipidemia affect the progression of atherosclerosis at a young age. Primary and secondary prevention of atherosclerotic cardiovascular disease is based on lifestyle modifications, especially dietary therapy, along with physical activity prescription, which should be applied as early as possible in children at risk. A lipid-lowering diet is considered the cornerstone of treatment, because of its effectiveness and safety in childhood. Dietary goals for children and adolescents with dyslipidemia are provided by the two-stage dietary approach of the Cardiovascular Health Integrated Lifestyle Diet (CHILD), which is composed of a two-step diet: CHILD-1 and, if the problem persists after 3 months, CHILD-2. Although dietary recommendations are well defined, traditional dietary assessment tools are unable to determine adherence to cholesterol-lowering diets. To fill this gap, a simplified 10-point Healthy Eating Assessment Tool (HEAT) was developed to assess a patient’s overall dietary quality and behaviors during time-limited individual counseling sessions [6]. The single-center prospective cross-sectional study by DiLauro et al. found significant correlations between HEAT scores and specific dietary intake variables included in the CHILD-2; the achievement of CHILD-2 recommendations, especially regarding the fat percentage daily calories; and associations between specific markers of adiposity [6]. The authors concluded that the HEAT may be useful in the assessment of dietary goals in children, particularly to identify those children with suboptimal dietary patterns that may put them at risk of obesity and dyslipidemia and who might benefit from targeted dietary counseling.

Childhood cancer survivors represent another fragile and growing population where cardiovascular health is an emerging issue. Since 1975, the rate of new cancer diagnoses among children and adolescents has slightly increased, whereas an overall reduction in mortality has been seen, leading to an increasing number of cancer survivors [7,8]. On the other hand, many antineoplastic therapies affect the cardiovascular system with short- and long-term complications, including congestive heart failure, myocardial infarction, pericardial disease and valvular abnormalities. In this scenario, customized nutritional interventions are crucial to improve dietary habits in children who survived cancer as a main strategy for secondary cardiovascular prevention. Guida et al. provide the reader with an extensive overview on the possible nutritional interventions in this population, focusing on the role of nutrition in the primary and secondary prevention of cardiovascular damage induced by antineoplastic therapies [9]. The authors discuss the burden of acute and chronic cardiotoxicity in childhood cancer survivors, dwelling on its potential mechanisms and alterations of the nutritional status, and finally provided a practical proposal to optimize the dietary habits of survivors and their families with a far-reaching list of nutrients that have exhibited a potential role in preventing chemotherapy-related cardiotoxicity. Properties, protective effects and related food sources are discussed for each compound.

In conclusion, as the editor and co-editors of this Special Issue, together with our collaborators, we thank the authors for their contributions. We are honored to present the papers collected in our Special Issue. Each paper is an outstanding contribution to the field of nutrition in pediatric cardiology. Several aspects of the relationship between nutrition and cardiovascular diseases are addressed, providing a great overview of this complex interplay. We thank the readers for their interest in our Special Issue and hope it meets all expectations.
